# Closed Genome Sequence of Aeromonas veronii Strain Hm21, an Isolate from the Medicinal Leech Hirudo verbana

**DOI:** 10.1128/MRA.00922-20

**Published:** 2020-10-15

**Authors:** Joerg Graf, Michael C. Nelson, Sophie M. Colston

**Affiliations:** a University of Connecticut, Department of Molecular and Cell Biology, Storrs, Connecticut, USA; University of Maryland School of Medicine

## Abstract

Aeromonas veronii strain Hm21 was isolated from the medicinal leech *Hirudo verbana* and is used for genetic studies. We present here the 4.71-Mbp genome with a 56-kb plasmid and identify the mutations present in strains commonly used for genetic engineering.

## Abstract (precis)

Aeromonas veronii strain Hm21 was isolated from the medicinal leech and is used for genetic studies. We present here the 4.71-Mbp genome with a 56-kb plasmid and identify the mutations present in strains commonly used for genetic engineering.

## ANNOUNCEMENT

Aeromonads are found in many aquatic environments and in pathogenic and beneficial associations with animals (1). One well-studied association is the digestive tract symbioses with leeches (2). Aeromonas veronii Hm21 was cultured from the digestive tract content of Hirudo verbana in 1996 by plating dilutions of the gut content on blood agar plates, incubating this overnight at 30°C, and maintaining it as a frozen stock culture (3). Antibiotic-resistant derivatives of this strain have been used in subsequent studies characterizing the molecular interactions between *A. veronii* and the host (4–6).

Previously, we published a genome based on Sanger sequencing, 454 pyrosequencing, and Illumina technology (7). This yielded a 4,684,957-bp genome comprising 75 contigs larger than 2 kb. We extracted the DNA using the MasterPure complete DNA and RNA purification kit (Epicentre, Madison, WI). For Illumina sequencing, NexteraXT libraries were prepared and sequenced on a MiSeq instrument (Illumina, San Diego, CA) (7). For PacBio sequencing, the unsheared DNA was used and the library prepared with the template kit 2.0 (PacBio, Menlo Park, CA), which was size selected using a BluePippin system (Sage Science, Beverly, MA). A single-molecule real-time (SMRT) cell was sequenced on the PacBio RS II platform using P5-C3 chemistry. A total of 60,113 reads with an *N*_50_ read length of 12,096 bp and a mean read score of 0.83 were assembled using the Hierarchical Genome Assembly Process (HGAP) 3 assembler with default parameters (8), yielding two contigs (4,710,355 bp [58.7% GC content] and 56,525 bp [59.7% GC content]) that were circularized manually. Using Illumina reads and default parameters, we used Trimmomatic for trimming the reads and adaptor trimming and breseq to polish the assemblies and detect mutations in the antibiotic-resistant derivatives (9, 10). The origin of replication was identified based on its proximity to *dnaA*, and the genome was rotated using Geneious R10 (Biomatters, Auckland, New Zealand). The genome was annotated using PGAP 4.12 for the NCBI submission (11). The closed genome contained 4,253 coding DNA sequences (CDS), 63 of which were on the plasmid, 10 complete rRNA operons, and 123 tRNAs.

The closed genome allowed us to examine repetitive features such as the rRNA operons. Five of the 10 16S rRNA genes were identical, but the others differed by up to 20 nucleotides. Such differences can lead to potentially misidentifying the species of *Aeromonas* (12). In this case, three out of seven sequences would have misidentified the species.

Several antibiotic-resistant derivatives of Hm21 that are used for genetic manipulations (3–6, 13) were analyzed. The genotypes of the rifampicin-resistant derivative, JG84, were *rpoB* S531F, a Δ21 deletion in an intragenic region at position 1648065, and *nrdD* K582N; the streptomycin-resistant derivative, JG1002, had the mutation *rpsL* K88R. Interestingly, JG304 (Hm21RS), a streptomycin-resistant derivative of JG84, lost the cryptic plasmid pHm21 while gaining *rpsL* K88R.

### Data availability.

This whole-genome shotgun project has been deposited at NCBI under the BioProject number PRJNA205862. The accession numbers are CP059396.1 and CP059397.1, and for the raw reads, the accession numbers are SRX8815181, SRX8815182, SRX8815183, SRX8815184, and SRX8815185. Strains are available from the corresponding author upon request.
